# Vitamin D Supplementation and Cognition in People with Type 2 Diabetes: A Randomized Control Trial

**DOI:** 10.1155/2019/5696391

**Published:** 2019-10-30

**Authors:** Mary A. Byrn, William Adams, Sue Penckofer, Mary Ann Emanuele

**Affiliations:** Loyola University Chicago, Chicago, IL, USA

## Abstract

**Aim:**

Type 2 diabetes increases the risk of cognitive decline which adversely impacts self-management of the disease. Evidence also supports a relationship between low serum 25(OH)D levels and poor cognition. The purpose of this trial was to assess vitamin D supplementation on cognitive executive functioning in persons living with type 2 diabetes.

**Methods:**

This was a double-blinded RCT where participants were randomized to receive either weekly vitamin D_3_ supplementation (50,000 IUs) or a matching comparator (5,000 IUs) for three months. The primary outcome was a battery of neuropsychological tests. Serum 25(OH)D was measured by liquid chromatography/tandem mass spectrometry. Repeated assessments of cognitive measures were collected over 12 weeks using alternative testing forms to minimize practice effects.

**Results:**

Thirty participants were randomized to either the low-dose allocation (*n* = 15) or the high-dose allocation (*n* = 15). Most participants were female (83%) and identified as Black (57%). For all cognition measures, there was no statistically significant finding between participants who received high-dose vitamin D supplementation and those who received low-dose supplementation. However, when assessing cognitive function in both groups over time, minimal improvement on the Symbol-Digits, the Stroop Interference Test, and the Trail Making Test Part B was observed.

**Conclusions:**

To our knowledge, this is the first randomized control trial to examine the effects of vitamin D supplementation on cognitive function in people with type 2 diabetes. However, no significant differences in cognitive outcomes between participants who received high-dose therapy and those who received low dose were found.

## 1. Introduction

Diabetes increases the risk of cognitive dysfunction. The incidence of dementia is 1.5 to 2.5 times higher in persons with diabetes than the general population [1]. There is evidence that cognitive decline significantly impacts the ability to self-manage diabetes [2]. Strategies to prevent cognitive decline in persons with diabetes have not been well studied. Interestingly, there is recent research suggesting the potential role of vitamin D in cognition and Alzheimer's disease [3]. One study reported that in persons who had vitamin D deficiency, the risk for all-cause dementia and Alzheimer's was doubled [4]. Vitamin D deficiency has been reported to negatively affect neuronal vitamin D receptors and adversely affect both growth factor signaling and neural activity [5, 6]. Therefore, providing vitamin D supplementation to improve cognition in persons with diabetes who are at a greater risk for cognitive dysfunction merits investigation.

Among individuals without diabetes, there is a significant association between low serum 25(OH)D and poor cognition. In fact, seven systematic reviews (or meta-analyses) have reported an association between low serum 25(OH)D and impaired cognition [7–13]. The majority of these syntheses were completed using observational designs (both cross sectional and longitudinal). Although there is evidence of an association between low serum 25(OH)D effects and poor cognition, clinical intervention studies have failed to associate increased serum 25(OH)D levels with improved cognitive outcomes [14]. However, one recent randomized controlled trial examined supplementation (4,000 vs. 400 IU daily) of vitamin D for 18 weeks in 82 healthy adults [15]. These authors reported that nonverbal (visual) memory seemed to improve from higher doses of vitamin D supplementation, more so for individuals with insufficient vitamin D levels at baseline (<75 nmol/L), but verbal memory and other cognitive domains did not [15].

For persons with diabetes, serum 25(OH)D levels are inversely associated with cognitive impairment suggesting that vitamin D could be a potential protective factor [16]. The effects of vitamin D supplementation on cognition in patients with type 2 diabetes, who are at a greater risk for poor cognition, have not been done and are a unique contribution of this study. The purpose of this study was to determine if vitamin D supplementation would improve cognition in people with type 2 diabetes. A randomized controlled trial was conducted to determine the effects of vitamin D_3_ supplementation in persons with type 2 diabetes who demonstrate at least some cognitive impairment. Persons were randomized to receive either weekly vitamin D_3_ supplementation (50,000 IUs) or a matching comparator (5,000 IUs) for a period of three months.

## 2. Methods

### 2.1. Patients

Patients with a diagnosis of type 2 diabetes were recruited between September 2015 and June 2018 by advertising the study through mailed letters, flyers in health clinics and local business, and calling eligible patients from a previous study. These recruitment methods have been used in previous research [17]. Patients were included in the study if they had a diagnosis of type 2 diabetes, a subjective or objective complaint of poor cognition, serum 25(OH)D level less than 32 ng/mL, a systolic blood pressure ≤ 160 and diastolic blood pressure ≤ 100, and currently being treated by a healthcare provider. Patients were excluded if they had a diagnosis of hypercalcemia (calcium ≥ 10.0), severe complications of diabetes (amputation, blindness, and dialysis), reduced kidney function (GFR < 60, creatinine > 1.2), a history of kidney stones (unless origin known to be not calcium), malabsorption problems, or history of stroke, TIA, or lacunar infarct. Participants were also excluded if they presented with significant depressive symptoms (CESD ≥ 16), a history of bipolar depression or psychosis, loss of consciousness greater than 5 minutes, or a current alcohol or substance use disorder. Further, participants were excluded if they were breast feeding, pregnant, or planning to become pregnant, and they were excluded if their HbA1c > 13%. The use of cholinesterase inhibitors was exclusionary as was a new prescription for any anxiolytic within the past 12 months; for patients currently using an anxiolytic, the dose had to remain stable for at least 12 months with no planned dose change while participating in the trial. Finally, patients were also excluded if they were taking high-dose steroids, vitamin D supplements in the past 2 months, or more than 1,200 mg of calcium per day; concomitant supplements known to affect cognition were also exclusionary including kava kava, St. John's Wort, and Ginkgo biloba.

### 2.2. Study Protocol

In this double-blinded, randomized, active-comparator controlled trial (Clinicaltrials.gov NCT02416193), patients received either high-dose (50,000 IU) vitamin D (cholecalciferol) therapy weekly (treatment) or low-dose (5,000 IU) cholecalciferol weekly for 12 weeks. A pharmacist prepared the dosing of vitamin D according to a randomized block schedule. Patients and researchers were blinded to treatment assignment. This study was branded as the THINK-D study under an investigational new drug number (IND 126491). Also, the study was approved by the institutional review board where data was collected.

Phone screening was used to establish interest in the study and assess initial eligibility to participate in the trial. The Center for Epidemiologic Scale (CES-D) was administered to ensure participants were free from severe depressive symptoms, and two telephone-based cognitive assessments were administered (the Controlled Oral Word Association Test and Animal Naming Test) to assess for objective cognitive impairment. Once patients completed the phone screen and all sampling criteria were met, they were scheduled for the baseline visit. Participants completed the baseline and final visits at two clinical sites within the same institution. Participants were able to choose the site that was closest and most convenient for them. At the first baseline visit, informed consent was obtained and participant's capacity to give written informed consent was evaluated. Once patients signed informed consent, cognitive tests (detailed below) were administered, blood was drawn, quality of life and functioning questionnaires were completed (detailed below), and anthropometric measures were completed. Once enrollment criteria were met, the participant attended the second visit where the vitamin D supplement was dispensed. Prior to taking the initial dose, the cognitive functioning tests were administered again in order to wash out any practice effects.

At the second visit, the possible side effects of the vitamin D supplement, the weekly medication administration schedule, and all subsequent contacts (phone and scheduled visits) were reviewed. After six weeks of therapy, a member of the research team called the participant to inquire about medication compliance, inquire about adverse side effects, and screen for cognitive function using the Controlled Oral Word Association Test and Animal Naming Test. After 12 weeks of therapy, participants returned for their final visit. At this visit, the exact protocol from baseline visit (i.e., the cognitive functioning tests, blood work, questionnaires, and anthropometric measures) was followed. A member of the research team inspected the study medication bottle to estimate drug accountability by counting remaining capsules. To minimize attrition and enhance compliance, retention strategies included free parking and compensation in a stepped manner: twenty dollars ($20) at the first baseline visit, $25 at the second visit, and $30 at the third visit. After the patients finished all three visits, they received a letter detailing the results of their HbA1c and serum 25(OH)D results. Patients also received a letter notifying them of study findings and gratitude for study participation.

### 2.3. Laboratory and Anthropometric Measures

Serum 25(OH)D was measured by liquid chromatography/tandem mass spectrometry (LC-MS/MS) (Quest Diagnostics). This method provides a total 25 hydroxyvitamin D (25 OH-D), which includes 25(OH)D_2_ and 25(OH)D_3_. Calcium was measured using a cardiometabolic profile (Quest Diagnostics). Due to different equipment at each site, two accepted methods of hemoglobin A1c were used: one site used the DCA Vantage Analyzer (Siemens Healthcare Diagnostics) which uses a fingerstick, and the other used venipuncture with samples sent to Quest Diagnostics. Most importantly, each participant had the same method used for the baseline and final visit. The Block Calcium and Vitamin D Screener was utilized to measure dietary intake of vitamin D and calcium [18]. Body weight was measured to the nearest 0.1 kg using a Healthometer Professional scale. Height was measured in centimeters using a stadiometer. Body mass index was calculated as the ratio of weight over height squared.

### 2.4. Cognition Measures

The primary outcome was to compare change in executive functioning between treatment groups. Executive functioning assesses individuals' cognitive control including their attention, inhibition, working memory, and cognitive flexibility [19–21]. For this domain, we assessed participants' verbal fluency using the Controlled Oral Word Association Test [22] and attention as well as speeded processing using the Symbol-Digit Modalities Test [23] and Stroop Color and Word Test (interference condition) [24]. We also included a measure of working memory using the Letter-Number Sequencing Test (from the Wechsler Adult Intelligence Scale-III) [25] and set-shifting ability using the Trail Making Test Part B [26]. A secondary goal of the study was to compare change in language and memory functioning between treatment groups. This domain was assessed using the Hopkins Verbal Learning Test [27] and Semantic Fluency Test [28]. Finally, to meet inclusion criteria, participants had to report either subjective complaints of poor cognition or objectively demonstrate poor premorbid intellect by scoring at least one standard deviation below average on the Wide Range Achievement Test-IV Reading subtest [29].

Except for the Letter-Number Sequencing Test, raw scores for all *executive functioning* assessments as well as the semantic fluency assessment were standardized as *Z*-scores (*μ* = 0, SD = 1) using age-adjusted performance metrics from a large healthy normative population; raw scores from the Letter-Number Sequencing Test were standardized as scaled scores (*μ* = 10, SD = 3) using age-adjusted normative data provided by Wechsler [25]. And raw scores on the Hopkins Verbal Learning Test were standardized as *t*-scores (*μ* = 50, SD = 10) using normative data provided by Brandt and Benedict [30]. For all assessments, higher scores indicate superior performance. When available from the test publisher, psychometrically equivalent alternative forms were used at each visit to minimize practice effects.

### 2.5. Quality of Life, Mood, and Functioning Measures

Baseline physical activity was assessed using the Godin Leisure-Time Exercise Questionnaire [31]. As described by Godin [31], scores less than 14 points on this assessment indicate insufficient physical activity while scores of 24 or higher indicate sufficient physical activity; other scores are categorized as being moderately active. Further, baseline mood was assessed using the CES-D and PHQ-9 assessments. Scores on the CES-D range from 0 to 60 points (where higher scores indicate greater depressive symptoms), and scores on the PHQ-9 range from 0 to 19 points (where higher scores indicate worsening mood).

Change in social adjustment from baseline to end of therapy was assessed using the Revised Social Adjustment Scale Self-Report (SAS-SR). As described by Weissman, scores on the SAS-SR are scaled as *t*-scores (*μ* = 50, SD = 10) where higher scores indicate worse social adjustment [32]. Change in diabetes self-care was assessed using the Revised Self Care Inventory (SCI-R), where scores are scaled as a percentage (min = 0, max = 100) with higher scores indicating better self-care [33]. We also assessed change in diabetes-related emotional distress from baseline to end of therapy using the Problem Areas in Diabetes (PAID) assessment. As described by Polonsky et al., the PAID is scored as an index (min = 0, max = 100) with higher scores indicating worse distress [34].

Change in work productivity was assessed using the Endicott Work Productivity Scale (EWPS) which, as described by Endicott and Nee, is an index (min = 0, max = 100) where higher scores indicate worse performance at work [35]. Further, change in sleep functioning was assessed using the Pittsburg Sleep Quality Index (PSQI). As described by Buysse et al., the PSQI is an index (min = 0, max = 21) where higher scores indicate greater sleep impairment [36]. Finally, change in quality of life and social support was assessed using the Medical Outcomes Study (MOS) survey which, as described by Sherbourne and Stewart, is a percentage (min = 0, max = 100) where higher scores indicate better social support [37]; change in stress was assessed using the Perceived Stress Scale (PSS) which, as described by Cohen et al., is an index (min = 0, max = 40) where higher scores indicate increased stress [38].

#### 2.5.1. Statistical Methods: Power

A meta-analysis based on a sample size of *N* = 7,688 by Etgen et al. was used to determine a sample size with suitable power for this trial [7]. In their study, individuals with insufficient vitamin D were nearly three times (95% CI: 1.91 – 3.00) more likely to experience executive dysfunction than individuals who were vitamin D sufficient. Our study was powered to test the null hypothesis that performance on a battery of executive functioning tests would be equal between individuals receiving high-dose vitamin D therapy and those receiving low-dose vitamin D therapy. These executive function scores were assumed to be standard scores (*μ* = 100, SD = 15).

With a proposed total sample size of 62 subjects assigned to the 50,000 IU and 5,000 IU dose treatment arms using a 1 : 1 allocation, the study would have a power of 81.1% to yield a statistically significant result. This computation assumed a two-sided alpha level of 0.05 that the mean difference between the two groups was less than one standard deviation (mean difference = 11.0) which corresponds to the expected means of *μ* = 96 for the 50,000 IU group and *μ* = 85 for the 5,000 IU dose group and that the common within-group standard deviation was one standard deviation (or 15 points). This effect was selected as the smallest effect that would be important to detect, in the sense that any smaller effect would not be of clinical or substantive significance. In order to account for attrition, 80 participants were planned to enroll into the study. At the end of three years, the study was halted prematurely due to slow recruitment and expiration of funding. During this timeframe, a total of 30 individuals were randomized.

#### 2.5.2. Statistical Methods

Summary frequencies are reported by treatment allocation for all nominal baseline characteristics, including sex, race, ethnicity, marital status, season of first dose, and physical activity level. Summary statistics are similarly reported as mean with standard deviation for normally distributed demographics (i.e., age, body mass index, blood pressures and heart rate, and CES-D score), while median with interquartile range is reported for other summary measures including the PHQ-9 score, WRAT-IV reading subscore, years of education, and laboratory values (i.e., vitamin D level, HbA1c, glucose, creatinine, and calcium).

Regarding performance on the *executive functioning* assessments, linear mixed-effects models were used to estimate the mean change in participants' standardized scores as a function of elapsed time since baseline, treatment assignment, and their interaction. In this study, patients could contribute multiple scores to the analysis and random intercepts were allowed for each patient to account for their within-subject correlation using an unstructured covariance structure. Regarding model fit, linearity and normality were assessed using residual and QQ plots, respectively, while outliers were assessed using box plots. When the interaction was not significant, it was removed from the model to estimate the average mean difference in performance between the two treatment groups while controlling for elapsed time since baseline. A similar approach was used to assess performance on assessments of *language and memory* functioning.

Regarding change in *quality of life* and *functioning* self-report measures (i.e., the SAS-SR, SCI-R, PAID, EWPS, PSQI, MOS, and PSS), each participant's baseline score was subtracted from his/her week 13 score. Subsequently, an independent sample *t*-test was used to compare this change score between participants assigned to the high dose versus those assigned to the low dose. In these comparisons, the distribution of survey scores was assessed for normality using QQ plots and for outliers using box plots. When survey scores were not normally distributed, an exact version of the Wilcoxon rank-sum test was used to confirm parametric conclusions.

Finally, summary frequencies are reported for all reported adverse events during the trial by treatment allocation. This includes the number of affected individuals at risk for the adverse event as well as the number of times the adverse event was reported. All analyses were completed by the trial Biostatistician (WA) using SAS version 9.4 (Cary, NC).

## 3. Results

### 3.1. Participant Characteristics

**Figure 1:**
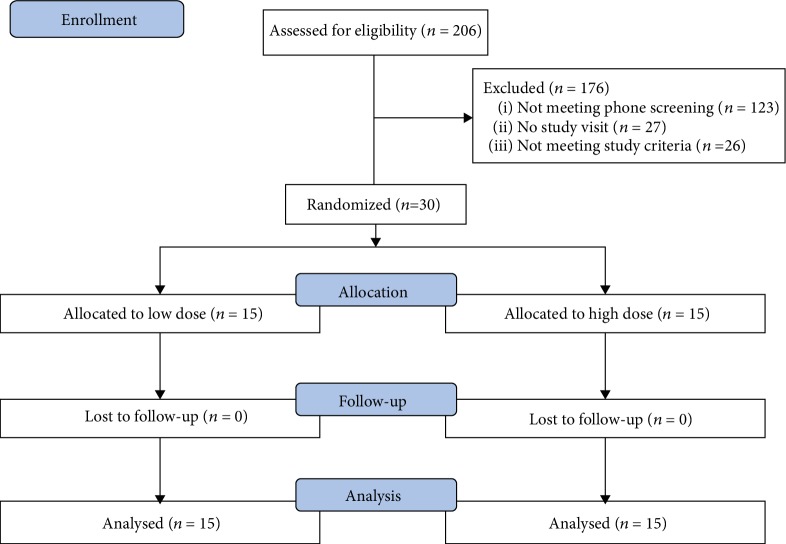
CONSORT diagram.

Of the 206 individuals assessed for eligibility, exactly 30 were randomized to either the low-dose allocation (*n* = 15) or high-dose allocation (*n* = 15) (see Figure 1).  Table 1 reports patient characteristics among those randomized to a treatment allocation. Most participants were female (83%) with a mean age of 55.71 (SD = 9.74) years. The majority were identified as Black (57%) with another 30% identifying as White; one individual was identified as Asian (3.3%) and another identified as Native Hawaiian (3.3%). Two individuals were identified as more than one race (6.7%). Regarding education, most had more than 12 years of education (*Mdn* = 14, IQR: 13 – 15). The majority were married (47%) with the remainder reporting that they were divorced (23%) or never married (20%); two individuals were widowers (6.7%), and one participant reported being separated (3.3%).

Regarding baseline health measures, the average body mass index was obese at 37.32 kg/m^2^ (SD = 8.11) though, as measured by the Godin Leisure-Time Exercise Questionnaire, the majority (43%) reported being active; another 20% reported being moderately active while 37% reported insufficient physical activity. The average systolic blood pressure was 132.93 mmHg (SD = 16.45) with an average diastolic blood pressure of 73.20 mmHg (SD = 10.70), which was elevated and classified as stage 1 hypertension according to the recently released hypertension guidelines [39]. Regarding baseline laboratory assessments, the median HbA1c was 7.15 (IQR: 6.30 – 7.70), median glucose was 134 mmol/L (IQR: 107 – 165), median creatinine was 0.79 mg/dL (IQR: 0.69 – 0.88), and median calcium level was 9.50 mg/dL (IQR: 9.30 – 9.70); the median total vitamin D level was 24 ng/mL (IQR: 17 – 27) while the median serum vitamin D_3_ (25 OH-D) was 22 ng/mL (IQR: 16 – 27). The HbA1c levels are slightly above the recommended goal of 7.0% or less for type 2 diabetes [40], and the vitamin D levels are insufficient according to the Endocrine Society [41].

Regarding premorbid intellect and mood, most participants scored below average on the Wide Range Achievement Test Reading Subtest (*Mdn* = ‐0.97, IQR: -1.20 to -0.33) but reported well-adjusted mood scores as measured by the PHQ-9 (*Mdn* = 3, IQR: 2 – 5) and CES-D (*μ* = 8.43, SD = 4.32).

### 3.2. Executive Functioning

**Figure 2:**
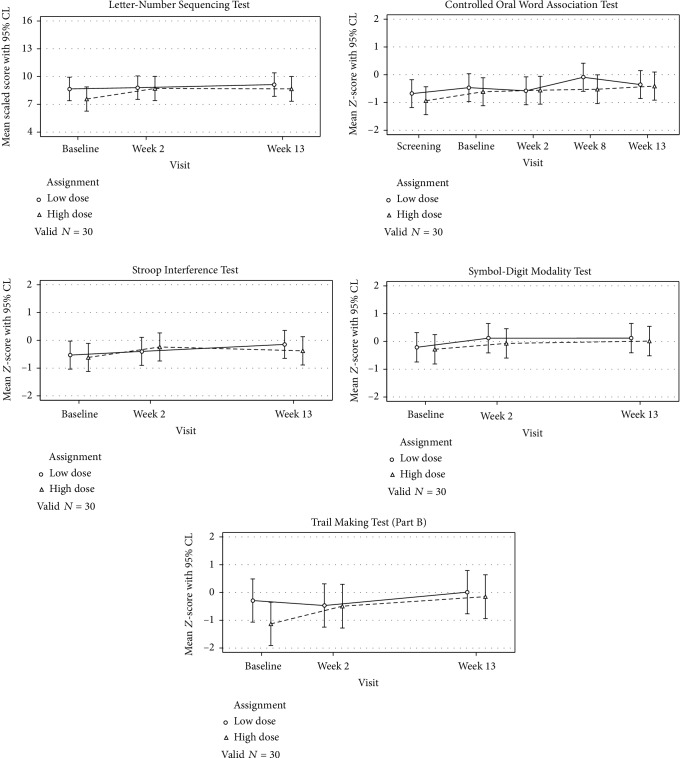
Executive functioning performance over time.

Figure 2 shows performance on assessments of executive functioning between those assigned to the high-dose therapy versus low-dose therapy over time, and Table 2 reports summary scores for each assessment by treatment allocation and time. For all five assessments, the association between treatment assignment and performance did not depend on elapsed time from baseline (all interaction *p* > 0.05). That is, scores for both treatments tended to either remain flat (i.e., on the COWAT and Letter-Number Sequencing assessments) or improve (i.e., on the Symbol-Digit Modality, Stroop Interference, and Trail Making Test Part B assessments) over time with no meaningful difference between groups. Indeed, after removing the interaction and controlling for elapsed time, there remained no significant difference between the two cohorts on the Symbol-Digit Modality Test (*M*_diff_ = ‐0.12, 95% CI: -0.84 – 0.60; *p* = 0.74), the Controlled Oral Word Association Test (*M*_diff_ = ‐0.17, 95% CI: -0.82 – 0.49; *p* = 0.62), the Stroop Interference Test (*M*_diff_ = ‐0.05, 95% CI: -0.71 – 0.61; *p* = 0.88), Part B of the Trail Making Test (*M*_diff_ = ‐0.35, 95% CI: -1.37 – 0.67; *p* = 0.49), or Letter-Number Sequencing Test (*M*_diff_ = ‐0.55, 95% CI: -2.19 – 1.10; *p* = 0.51).

Regarding overall performance over time, it is important to note that alternative forms were used (when available) to minimize practice effects. Still, controlling for treatment assignment, Symbol-Digit Modality scores improved from baseline by approximately 0.27 (95% CI: 0.09 – 0.45) standardized units by week 2 (*p* = 0.003) and by 0.31 (95% CI: 0.13 – 0.49) standardized units by week 13 (*p* = 0.001). Conversely, Controlled Oral Word Association scores remained constant from baseline to week 2 (*M*_diff_ = ‐0.03, 95% CI: -0.25 – 0.19; *p* = 0.79), but nominally improved by week 8 (*M*_diff_ = 0.24, 95% CI: 0.01 – 0.48; *p* = 0.04); COWAT scores showed no difference from baseline by the end of treatment (*M*_diff_ = 0.15, 95% CI: -0.07 – 0.38; *p* = 0.18). Performance on the Stroop Interference Test improved from baseline by week 2 (*M*_diff_ = 0.26, 95% CI: 0.01 – 0.50; *p* = 0.04) and week 13 (*M*_diff_ = 0.32, 95% CI: 0.07 – 0.56; *p* = 0.01), while scores on the Trail Making Test Part B remained flat between baseline and week 2 (*M*_diff_ = 0.22, 95% CI: -0.18 – 0.62; *p* = 0.27) before showing nominal improvement by week 13 (*M*_diff_ = 0.64, 95% CI: 0.24 – 1.04; *p* = 0.002). Regarding performance on the Letter-Number Sequencing Test, performance remained flat over all study visits even after controlling for treatment assignment (overall *p* = 0.07).

### 3.3. Language and Memory Functioning

**Figure 3:**
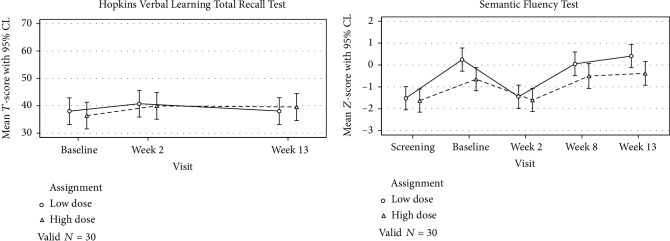
Language and memory functioning performance over time.

Figure 3 shows performance on assessments of language and memory functioning between those assigned to the high dose versus low dose over time. For both assessments, the association between treatment assignment and performance did not depend on elapsed time from baseline (both interaction *p* > 0.05). That is, scores for both treatments were generally flat over time with no meaningful difference between groups. Indeed, after removing the interaction and controlling for elapsed time, there remained no significant difference between the two treatments on the Hopkins Verbal Learning Test (*M*_diff_ = ‐0.34, 95% CI: -6.33 – 5.65; *p* = 0.91) or the Semantic Fluency Test (*M*_diff_ = ‐0.50, 95% CI: -1.07 – 0.07; *p* = 0.09).

As before, we used alternative forms to minimize practice effects and noted that overall scores on the Hopkins Verbal Learning Test remained flat over time even after controlling for treatment assignment (overall *p* = 0.12). Conversely, controlling for treatment assignment, scores on the Semantic Fluency Test were random: performance remained flat from baseline to week 8 (*M*_diff_ = ‐0.02, 95% CI: -0.44 to 0.39; *p* = 0.92) and week 13 (*M*_diff_ = 0.22, 95% CI: -0.18 to 0.62; *p* = 0.28), but there was a significant drop in performance between baseline and week 2 (*M*_diff_ = ‐1.33, 95% CI: -1.72 to -0.93; *p* < 0.001).

### 3.4. Self-Report Surveys

Table 3 reports change in social adjustment (using the SAS-SR), diabetes self-care (using the SCI-R), diabetes-related emotional distress (using the PAID), work productivity (using the EWPS), sleep functioning (using the PSQI), quality of life and social support (using the MOS), and stress (using the PSS) from baseline to end of treatment. In this sample, there was no association between treatment assignment and change in SAS-SR (*p* = 0.15), SCI-R (*p* = 0.36), PAID (*p* = 0.37), EWPS (*p* = 0.16), PSQI (*p* = 0.57), MOS (*p* = 0.75), or PSS (*p* = 0.79).

### 3.5. Adverse Events

Regarding safety and tolerability, 21 subjects experienced adverse events: 12 in the low-dose allocation and nine in the high-dose allocation. In the low-dose allocation, the most frequent complaint was nausea (20%). Conversely, in the high-dose allocation, the most frequent complaint was a cold virus (27%) (see Table 4).

## 4. Discussion

To our knowledge, this is the first randomized controlled trial to examine the effects of vitamin D supplementation on cognitive function in people with type 2 diabetes. There were no significant differences in improvement in cognitive functioning between participants who received high dose (50,000 IU weekly) compared to those who received low dose (5,000 IU weekly). Although, no significant difference was found between groups, there was some significant improvement over the three months of the study for all study participants. The three cognitive tests for executive function that improved significantly from baseline to week 13 for all participants were Symbol-Digit, Stroop Interference, and Trail Making Part B. Interestingly, mean serum 25(OH)D levels increased to within the normal range for both groups. However, because the study lacked a true control group given a placebo, it is not possible to conclude if the low dose of vitamin D supplementation (5,000 IU weekly) was sufficient to improve executive functioning in people with type 2 diabetes.

Pettersen [15] and Dean et al. [42] completed randomized control trials to study the impact of vitamin D supplementation on cognition in healthy adults. Pettersen randomized participants to receive 4,000 IU or 400 IU of vitamin D (cholecalciferol) daily. A battery of cognitive tests was used to measure cognitive functioning including: Symbol-Digit Modalities Test, phonemic fluency, One-Touch Stockings of Cambridge, digit span forward and backward, verbal recognition memory, pattern recognition, Paired-Associate Learning, and Spatial Working Memory [15]. Pettersen found that participants with insufficient serum 25(OH)D levels at baseline and in the high-dose group had significant improvement in nonverbal visual memory as measured by the pattern recognition memory (*p* = 0.026). Also, participants receiving the low-dose vitamin D supplementation improved significantly in verbal memory (*p* = 0.054) [15]. Although, Pettersen reported significant findings using the pattern recognition memory test for participants in the high-dose group and the verbal recognition memory test for participants in the low-dose group, the current study did not use either of those cognitive exercises making comparisons between study findings impossible. However, the current study and Pettersen both used Symbol-Digit as a measure for executive functioning, but only the current study reported a significant improvement on that test in all participants (both high dose and low dose).

Dean et al.[42] randomized participants to receive either 5,000 IU of vitamin D (cholecalciferol) daily or placebo. To measure cognitive functioning, three tests were used: N-Back, stop-signal task response inhibition, and set shifting task [42]. Dean et al. [42] reported no significant improvements in cognitive functioning in the group receiving vitamin D supplementation compared to the group receiving placebo. The lack of significant findings was similar to the current study findings; however, direct comparisons between this study and the current study are difficult given that cognition was measured differently.

Although the present study did not find a significant difference in improvement of cognition between high-dose and low-dose groups, cognitive functioning improved in both groups. A true improvement on cognition related to vitamin D supplementation should not be ruled out due to the small sample size and lack of a true placebo group. Given that both groups had an increase in serum 25(OH)D levels to the above recommended level and both groups saw slight improvements in cognition, the positive effect of vitamin D supplementation on cognition is still plausible. Two review studies reported that even a small increase of 10 ng/mL in serum vitamin D levels was sufficient to see improvements in depression [43] and colorectal cancer and chronic kidney disease [44]. However, larger randomized control trial studies with a placebo group would be needed to test this hypothesis.

The current study had some limitations. One limitation was the small sample size. The small sample size may not have been powered appropriately to detect changes between groups in cognitive improvement. The inclusion criteria for serum vitamin D levels were set at less than 32 ng/mL; therefore, people with insufficient and borderline normal serum vitamin D levels were enrolled in the study. This may have diluted the ability for improvements in cognition to be documented. Also, the lack of a true placebo group and both groups improving significantly on mean serum 25(OH)D levels make conclusions related to the impact of vitamin D on cognition difficult. The study length of supplementation for only three months may not have been adequate time to measure true improvements in cognition. Although the study was designed to diminish the practice effects that occur with cognitive testing, the impact of practice effects may still have had an impact on the improvement of cognitive tests from baseline to the 3-month measures.

Despite the limitations, the study included many strengths, such as the double-blind randomized control trial design. Also, the sample was comprised of only participants with a subjective complaint of poor cognition and at risk for cognitive decline given that all participants had type 2 diabetes. The sample was very diverse including 57% Black, which represents the population of people that are at an increased risk for diabetes, low serum 25(OH)D, and cognitive decline [45, 46]. Another strength of the current study was the use of a battery of cognitive tests to measure cognition. Many studies use the Mini-Mental State Exam as a global measure of cognition; however, there have been reported ceiling and floor effects with that measure [47]. Using a battery of tests allowed for even small improvements in cognition over a short period of time to be detected and allowed for many aspects of cognition (executive functioning, language, and memory) to be studied. The study had a very low attrition rate (100% completion) and a high compliance with weekly dosing (only two participants reported missing a single dose or one week of therapy).

## 5. Conclusion

Although our study does not support the findings that vitamin D supplementation improves cognition in a high risk population of people who are at risk for cognitive decline, observational studies and animal studies suggest an association. Larger randomized control trials over a longer time should be conducted before conclusions are made on the benefits or lack thereof of vitamin D supplementation on cognition. Future trials should include strengths from this study including enrolling a diverse sample of participants at risk for low serum 25(OH)D and poor cognition. Also, the use of a battery of cognitive tests and appropriate dosing of vitamin D supplementation for participants with low serum 25(OH)D levels should be utilized. Future trials may want to consider enrolling participants with serum vitamin D levels less than 20 ng/mL and using a true placebo group to have the best chances of determining the effects of vitamin D supplementation on cognition. Future research will improve the knowledge to determine if cost effective preventative treatments, such as vitamin D supplementation, would be a beneficial addition to the treatment plan of patients with type 2 diabetes who are at risk for dementia.

## Figures and Tables

**Table 1:** Participant characteristics.

	Low dose (*n* = 15)	High dose (*n* = 15)	Total (*N* = 30)
Sex			
Male	1 (6.7%)	4 (27%)	5 (17%)
Female	14 (93%)	11 (73%)	25 (83%)
Mean age (SD)	55.62 (10.14)	55.80 (9.68)	55.71 (9.74)
*Mdn* years of education (IQR)	14 (12 – 15)	14 (13 – 16)	14 (13 – 15)
Race			
White	5 (33%)	4 (27%)	9 (30%)
Black or African American	8 (53%)	9 (60%)	17 (57%)
Asian	0	1 (6.7%)	1 (3.3%)
Native Hawaiian or other Pacific Islander	1 (6.7%)	0	1 (3.3%)
Multiracial	1 (6.7%)	1 (6.7%)	2 (6.7%)
Ethnicity			
Not Hispanic or Latino	10 (67%)	13 (87%)	23 (77%)
Hispanic or Latino	4 (27%)	2 (13%)	6 (20%)
Unknown or not reported	1 (6.7%)	0	1 (3.3%)
Duration of diabetes			
<6 months	2 (13%)	0	2 (6.7%)
6-12 months	0	2 (13%)	2 (6.7%)
1-5 years	2 (13%)	2 (13%)	4 (13%)
5-10 years	6 (40%)	4 (27%)	10 (33%)
>10 years	5 (33%)	7 (47%)	12 (40%)
*Mdn* vitamin D levels in ng/mL (IQR)			
Total vitamin D			
Baseline	21 (15 – 25)	25 (19 – 28)	24 (17 – 27)
Week 13	27 (23 – 32)	53 (49 – 64)	42 (27 – 53)
Serum vitamin D_3_ (25 OH-D)			
Baseline	21 (13 – 25)	24 (18 – 28)	22 (16 – 27)
Week 13	27 (23 – 32)	53 (49 – 64)	42 (27 – 53)
Mean body mass index (SD)	40.44 (9.48)	34.20 (5.05)	37.32 (8.11)
Marital status			
Currently married	10 (67%)	4 (27%)	14 (47%)
Separated	0	1 (6.7%)	1 (3.3%)
Widowed	2 (13%)	0	2 (6.7%)
Divorced	2 (13%)	5 (33%)	7 (23%)
Never married	1 (6.7%)	5 (33%)	6 (20%)
Season of first dose			
Fall	5 (33%)	5 (33%)	10 (33%)
Winter	3 (20%)	6 (40%)	9 (30%)
Spring	5 (33%)	1 (6.7%)	6 (20%)
Summer	2 (13%)	3 (20%)	5 (17%)
Mean vitals (SD)			
Systolic blood pressure	134.73 (16.33)	131.13 (16.95)	132.93 (16.45)
Diastolic blood pressure	73.47 (10.99)	72.93 (10.79)	73.20 (10.70)
Heart rate	73.27 (12.67)	76.00 (12.86)	74.63 (12.62)
*Mdn* laboratory values (IQR)			
HBA1C (mmol/mol)			
Baseline	7.10 (6.30 – 7.50)	7.20 (6.20 – 7.80)	7.15 (6.30 – 7.70)
Week 13 (*N* = 29)	7.10 (6.30 – 7.70)	6.90 (6.00 – 8.60)	7.10 (6.20 – 7.70)
Glucose (mmol/L)			
Baseline	120 (95 – 160)	142 (116 – 214)	134 (107 – 165)
Week 13	126 (98 – 138)	128 (100 – 195)	127 (100 – 160)
Creatinine (mg/dL)			
Baseline	0.74 (0.66 – 0.90)	0.83 (0.70 – 0.88)	0.79 (0.69 – 0.88)
Week 13	0.78 (0.68 – 0.81)	0.80 (0.68 – 0.92)	0.79 (0.68 – 0.87)
Calcium (mg/dL)			
Baseline	9.40 (9.30 – 9.60)	9.60 (9.40 – 9.90)	9.50 (9.30 – 9.70)
Week 13	9.50 (9.20 – 9.70)	9.70 (9.40 – 9.90)	9.50 (9.40 – 9.80)
Godin activity level			
Insufficiently active	5 (33%)	6 (40%)	11 (37%)
Moderately active	3 (20%)	3 (20%)	6 (20%)
Active	7 (47%)	6 (40%)	13 (43%)
*Mdn* WRAT-4 Reading *Z*-score (IQR)	-1.00 (-1.20 to -0.33)	-0.93 (-1.20 to -0.20)	-0.97 (-1.20 to -0.33)
*Mdn* PHQ-9 score (IQR)	2 (1 – 4)	4 (3 – 7)	3 (2 – 5)
Mean CES-D score (SD)	7.13 (3.46)	9.73 (4.80)	8.43 (4.32)

*Note*: unless otherwise stated, valid *N* = 30 for all demographics. SD = standard deviation of the mean. *Mdn* = median. IQR = interquartile range. ng/mL = nanograms per milliliter. PHQ-9 = Patient Health Questionnaire 9. CES-D = Center for Epidemiologic Studies Depression Scale.

**Table 2:** Summary statistics for cognitive test scores.

			Assignment								
			Low dose			High dose			Total		
			*n*	*M*	SD	*n*	*M*	SD	*N*	*M*	SD
Symbol-Digit Modality *Z*-score	Event	Screening	0	.	.	0	.	.	0	.	.
		Baseline	15	-.21	1.01	15	-.28	1.26	30	-.25	1.12
		Week 2	15	.12	.91	15	-.07	1.10	30	.02	1.00
		Week 8	0	.	.	0	.	.	0	.	.
		Week 13	15	.12	.82	14	.05	1.20	29	.08	1.01

Verbal fluency *Z*-score	Event	Screening	15	-.68	1.28	15	-.94	.80	30	-.81	1.06
		Baseline	15	-.46	1.13	15	-.61	.78	30	-.54	.95
		Week 2	15	-.58	.95	15	-.56	.98	30	-.57	.95
		Week 8	14	-.10	1.27	12	-.36	.81	26	-.22	1.07
		Week 13	15	-.35	1.10	14	-.37	.93	29	-.36	1.00

HVLT total recall *T*-score	Event	Screening	0	.	.	0	.	.	0	.	.
		Baseline	15	38.00	7.01	15	36.40	10.47	30	37.20	8.79
		Week 2	15	40.73	8.48	15	39.93	11.51	30	40.33	9.94
		Week 8	0	.	.	0	.	.	0	.	.
		Week 13	15	38.07	8.92	14	39.57	11.50	29	38.79	10.09

HVLT delayed recall *T*-score	Event	Screening	0	.	.	0	.	.	0	.	.
		Baseline	15	38.00	10.52	15	36.93	12.14	30	37.47	11.17
		Week 2	15	38.40	12.25	15	38.80	13.51	30	38.60	12.67
		Week 8	0	.	.	0	.	.	0	.	.
		Week 13	15	36.93	8.92	14	37.21	14.30	29	37.07	11.61

HVLT retention *T*-score:	Event	Screening	0	.	.	0	.	.	0	.	.
		Baseline	15	42.27	12.62	15	41.20	14.12	30	41.73	13.17
		Week 2	15	41.93	15.40	15	39.20	12.34	30	40.57	13.78
		Week 8	0	.	.	0	.	.	0	.	.
		Week 13	15	41.47	11.87	14	40.29	15.64	29	40.90	13.58

Stroop word reading *Z*-score	Event	Screening	0	.	.	0	.	.	0	.	.
		Baseline	15	-.78	.58	15	-.88	.78	30	-.83	.68
		Week 2	15	-.67	.46	15	-.76	.67	30	-.72	.57
		Week 8	0	.	.	0	.	.	0	.	.
		Week 13	15	-.56	.26	14	-.62	.90	29	-.59	.64

Stroop color naming *Z*-score	Event	Screening	0	.	.	0	.	.	0	.	.
		Baseline	15	-.73	.57	15	-.92	.59	30	-.82	.58
		Week 2	15	-.56	.61	15	-.73	.55	30	-.64	.58
		Week 8	0	.	.	0	.	.	0	.	.
		Week 13	15	-.38	.56	14	-.73	.72	29	-.55	.65

Stroop Interference *Z*-score	Event	Screening	0	.	.	0	.	.	0	.	.
		Baseline	15	-.53	.88	15	-.62	1.23	30	-.57	1.05
		Week 2	15	-.40	.97	15	-.24	1.06	30	-.32	1.00
		Week 8	0	.	.	0	.	.	0	.	.
		Week 13	15	-.15	.88	14	-.30	.96	29	-.22	.91

Trail Making Test Part A *Z*-score	Event	Screening	0	.	.	0	.	.	0	.	.
		Baseline	15	-.02	.91	15	-.15	1.58	30	-.09	1.27
		Week 2	15	-.02	.67	15	-.05	1.85	30	-.04	1.37
		Week 8	0	.	.	0	.	.	0	.	.
		Week 13	15	.05	.63	14	.17	1.47	29	.11	1.10

Trail Making Test Part B *Z*-score	Event	Screening	0	.	.	0	.	.	0	.	.
		Baseline	15	-.29	.82	15	-1.13	2.63	30	-.71	1.96
		Week 2	15	-.47	1.07	14	-.29	1.68	29	-.38	1.37
		Week 8	0	.	.	0	.	.	0	.	.
		Week 13	15	.01	.85	14	-.10	1.35	29	-.04	1.10

Letter-Number Sequencing Scaled Score	Event	Screening	0	.	.	0	.	.	0	.	.
		Baseline	15	8.67	1.50	14	7.57	3.96	29	8.14	2.95
		Week 2	15	8.80	1.32	14	8.71	2.87	29	8.76	2.17
		Week 8	0	.	.	0	.	.	0	.	.
		Week 13	15	9.13	2.53	13	9.00	1.87	28	9.07	2.21

Naming *Z*-score	Event	Screening	15	-1.52	.98	15	-1.63	.70	30	-1.57	.84
		Baseline	15	.25	1.03	15	-.65	.95	30	-.20	1.07
		Week 2	15	-1.45	1.16	15	-1.60	.84	30	-1.53	1.00
		Week 8	14	.06	1.45	12	-.52	1.01	26	-.21	1.28
		Week 13	15	.41	1.46	14	-.36	.95	29	.04	1.28

**Table 3:** Change in quality of life and functioning assessments.

	Valid N	Delta score (week 13—baseline)			*p*
		Mean difference	95% confidence interval		
			Lower	Upper	
SAS-SR	30	-3.93	-9.35	1.48	.15
SCI-R	30	4.31	-5.12	13.75	.36
PAID	30	3.08	-3.78	9.95	.37
EWPS	15	-5.89	-14.42	2.64	.16
PSQI	27	0.53	-1.36	2.42	.57
MOS	28	1.22	-6.55	8.99	.75
PSS	30	-0.53	-4.54	3.47	.79

*Note*: the mean difference and 95% confidence limits of the delta score (week 13—baseline) are shown for the high dose versus low dose. SAS-SR = Social Adjustment Scale Self-Report. SCI-R = Self-Care Index Revised. PAID = Problem Areas in Diabetes. EWPS = Endicott Work Productivity Scale. PSQI = Pittsburg Sleep Quality Index. MOS = Medical Outcomes Study Social Support Survey. PSS = Perceived Stress Scale.

**Table 4:** Adverse events by treatment allocation.

	Low dose (*n* = 15)		High dose (*n* = 15)	
	Number affected	Number of events	Number affected	Number of events
Cold virus	1 (6.7%)	1	4 (27%)	4
Short menstrual period	1 (6.7%)	1	0	0
Menstrual cramps	0	0	1 (6.7%)	2
Arm pain	1 (6.7%)	1	0	0
Toothache	0	0	1 (6.7%)	1
Nausea	3 (20%)	3	0	0
Constipation	1 (6.7%)	1	0	0
Cataracts	1 (6.7%)	1	0	0
Hypercalcemia	0	0	1 (6.7%)	1
Hemorrhoids	1 (6.7%)	1	0	0
Poor appetite	0	0	1 (6.7%)	1
Dark stools	1 (6.7%)	1	0	0
Asthma	0	0	1 (6.7%)	1
Sinus infection	0	0	1 (6.7%)	1
Strep throat	1 (6.7%)	1	0	0
Sore throat	1 (6.7%)	3	0	0
Fall	1 (6.7%)	1	0	0
Tiredness	1 (6.7%)	1	0	0
Muscle aches	0	0	1 (6.7%)	1
Goiter	0	0	1 (6.7%)	1
Arthritis	1 (6.7%)	1	1 (6.7%)	1
Foot pain	2 (13%)	2	0	0
Left-sided weakness	0	0	1 (6.7%)	1
Restlessness	0	0	1 (6.7%)	1
Shoulder pain	1 (6.7%)	1	0	0
Knee cyst	1 (6.7%)	1	0	0
Leg cramps	2 (13%)	2	0	0
Motor vehicle accident	1 (6.7%)	1	0	0
Hypertension	0	0	2 (13%)	2

## Data Availability

The data used to support the findings of this study are included within the article.
